# Improving City Water Quality through Pollution Reduction with Urban Floodgate Infrastructure and Design Solutions: A Case Study in Wuxi, China

**DOI:** 10.3390/ijerph191710976

**Published:** 2022-09-02

**Authors:** Lan Feng, Pan Hu, Haisen Wang, Ming-ming Chen, Jiangang Han

**Affiliations:** 1 College of Civil Engineering, Nanjing Forestry University, Nanjing 210037, China; 2 Ecological Complexity and Modeling Laboratory, Department of Botany and Plant Sciences, University of California, Riverside, CA 92521, USA; 3 College of Environment and Biology, Nanjing Forestry University, Nanjing 210037, China; 4 College of Engineering, University of Alabama, Tuscaloosa, AL 35401, USA; 5 Collaborative Innovation Center of Southern Modern Forestry, Nanjing Forestry University, Nanjing 210037, China; 6 National Positioning Observation and Research Station, Hongze Lake Wetland Ecosystem, Huaian 223100, China

**Keywords:** small floodgates, river network, design solutions, water environmental model, water pollution

## Abstract

Floodgate operation is one of the main forms of river regulation in the development and utilization of river basins. It changes the natural structure, flow process, and correlative environment of rivers. However, there is little analysis of the multiple impacts of small floodgate operation on the water environment in river networks and of the regulation patterns of urban floodgate infrastructure on pollution. In this paper, a one-dimensional hydrodynamic–water quality model, MIKE 11, was used, taking Wuxi’s two main pollutant indicators—the permanganate index (COD_Mn_) and ammonia nitrogen (NH_3_-N)—to simulate the water quality response of Wuxi’s river network based on different design solutions of urban floodgate infrastructure. The results show that among the three design scenarios, the order of the decreasing amplitude of the COD_Mn_ and NH_3_-N concentrations was as follows: 1.4 m design solution scenario > 2.1 m design solution scenario > 0.7 m design solution scenario. Meanwhile, under the 1.4 m scenario, the maximum decrease in the COD_Mn_ concentration reached 37.57%, and the maximum decrease in the NH_3_-N concentration reached 206%. In the entire river network system, the improvement in the water quality in the downstream area was significantly better than that in the upstream area. In addition, under the three scenarios of floodgate operation, the changes in pollutant concentrations during the flood season (June–September) were significantly lower than those during the dry season (October–February) and the flat water season (March–May). The research results can provide theoretical support and new ideas for future research on the ecological operation of small floodgates and related research on the water environment effect.

## 1. Introduction

The lack of trade-offs between environmental protection and human exploitation has brought about increasingly undesirable impacts on the water environment, resulting in its degradation and collapse [1]. Recent initiatives, including the United Nations Sustainable Development Goals (SDG), have highlighted the significance of sustainable management and protection in avoiding prominent detrimental impacts on the water environment. It is fundamental to regulate the responsibilities for protection and to engage stakeholders in co-management; thus, decision makers demand detailed maps of where to prioritize human activities and alleviate their adverse impacts [2]. In the last two decades, more attention has been poured into managing multi-objective floodgate or dam uses, especially in a river network area where human activities deeply jeopardize the aquatic environment [3,4]. However, this relationship remains a disappearing link in decision making because of the question of identifying the human activities that trigger aquatic habitat degradation [5]. Future research objectives should be focused on understanding and quantifying the relationship between human activities and the water environment [6].

Global warming is the primary cause of climate change, which has emerged as one of the most pressing environmental challenges of our day [7]. Temperature increases, severe weather, more frequent climatic events, and accelerated hydrological cycles on continental and global scales are all results of global climate change [8]. As a result, governments and experts around the world are very concerned and interested in learning more about the effects of climate change [9]. River floods are one of the most destructive types of natural disasters. In recent decades, flooding of various forms, including fluvial, pluvial, and coastal flooding, has resulted in increasingly severe catastrophes and gained public awareness. The average annual global cost of catastrophic floods is estimated at USD 104 billion [10]. It is anticipated that continued climate change, urbanization, and economic growth will result in more extreme flooding episodes [11,12]. The magnitude of floodgates and dams being built is growing as a result of changes in the intensity, frequency, and patterns of flooding events on watershed, continental, and global scales.

Enormous flood prevention facilities are being constructed on rivers throughout the world, such as in India [13], China [14], Canada [15], and Brazil [16]. While the environmental effects of large dams or floodgates are universally known, those of small floodgates or dams (≤15 m or ≤3 × 106 m^3^) have rarely been considered [17]. In view of the proliferation of flood prevention dams in the world’s river systems, a challenge appears in relation to their cumulative impacts on water environments. Endeavors to evaluate these flood prevention facilities’ cumulative environmental impacts suggested that a large quantity of small dams or floodgates may have an immeasurable impact on energy generation compared to large ones [18]. Thus, there is an urgent requirement to understand the multiple environmental impacts of small flood prevention development and to understand how these dams or floodgates might be better developed and managed.

Previous studies indicated that dams can negatively affect the function of riverine systems [19,20,21]. Dams have modified the biochemical processes in riparian ecosystems, estuaries, deltas, and water environments, which in turn affects vegetation, regional climate change, and human health throughout the world [22]. Of course, the adverse consequences of dams have always existed. Meanwhile, the benefits and risks of dams and floodgates have been extensively analyzed in the literature [23,24,25,26,27,28,29,30]. For instance, the benefits and risks fluctuate during the various periods of a project’s development. While the majority of the costs, including socio-economic and environmental aspects, are triggered in the early stages, plenty of the benefits accumulate across the lifespan of the project, such as increased hydropower production, better flood prevention terms, and water transfer balance [31].

Nevertheless, limited scholastic research has been conducted involving the positive aspects of small floodgates, especially in human activities. Despite the fact that some scientific attention has been paid to the benefits of flood prevention dams, this is usually within the framework of single dam or floodgate studies [13,21], and whereas the great quantity of studies focusing on the potential mutual gains of hydropower dams is continually increasing, not much has been said about flood prevention dams in a river network, which remain understudied except for some research that is already more than five years old.

Meanwhile, some attention has been paid to river water quality improvement by small floodgates and dams, and lots of preliminary studies have discussed the water transfer in China. For instance, Bu et al. [32] adopted the MIKE11 model to simulate the water ecological carrying capacity (WECC) under various inflow and river connectivity index scenarios, which provided insights into improving the WECC. Chen et al. [33] integrated a variety of models including MIKE11 and SWAT and developed a comprehensive modeling method for the research on the water quality response of non-point source discharge in complex urban–rural areas. Li et al. [34] established a hydrological hydrodynamic–water quality coupling model of the Shahe Reservoir Basin using MIKE and other models to analyze the migration and transformation laws of point and non-point source pollutants such as ammonia nitrogen (NH_3_-N), chemical oxygen demand (COD), and total phosphorus (TP), so as to provide technical support for the decision making of comprehensive restoration and improvement projects of river water environments. Xiong et al. [35] applied the MIKE model to carry out a series of water replenishment measures to improve river water quality, providing a new idea for water pollution control. Li et al. [36] used the MIKE model to simulate the regulation of the water level and water quality improvement, which provided a theoretical basis for the improvement of the water quality of urban river-type reservoirs. However, research on simulating the temporal and spatial characteristics of water body improvement under the different regulations of gates and dams is relatively scarce, especially for urban river network areas. In addition, whether there is an optimal regulation mode is also one of the important issues to be explored in this field.

This present study centers on the regulation patterns of urban floodgate infrastructure on pollution and identifies the human activities answerable for urban river preservation based on different design solutions. The goals were to (1) characterize the spatial–temporal distribution patterns of pollution under floodgates, (2) design regulation patterns of urban floodgate infrastructure on the grounds of human impacts, and (3) improve the ability of the spatial regulation to identify and prioritize flood control strategies.

## 2. Study Area

**Figure 1:**
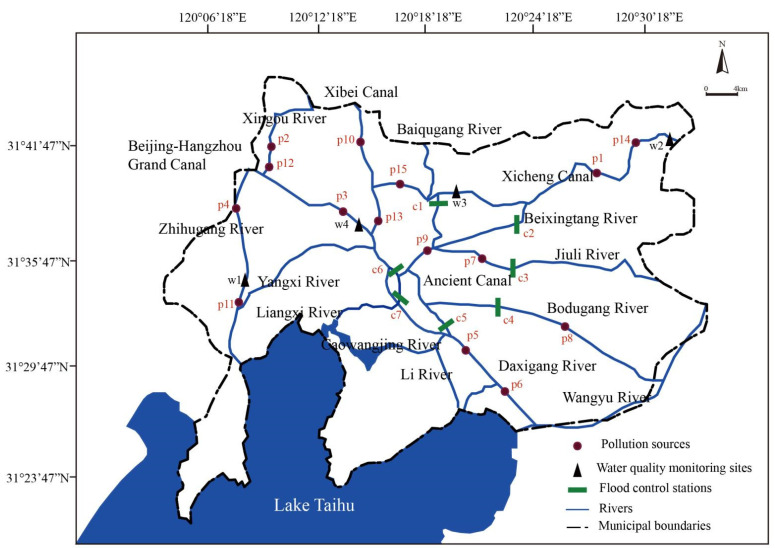
Research area and sampling points.

Wuxi (31°7′–32°2′ N and 119°33′–120°38′ E) is located on the north bank of Taihu Lake in the Yangtze River Delta of East China (Figure 1). It owns 1.7 billion m^3^ of the total water resources and has an average annual rainfall of 1048 mm, but water resources per capita are less than one sixth of the national average [37]. In the past 30 years, due to the rapid development of industrialization and urbanization, water resources in Wuxi city have been seriously declining. In June 2007, Wuxi attracted worldwide attention because of its regional water supply crisis caused by blue algae in Taihu Lake [38].

Wuxi is an archetypal example of a Chinese flooding area. According to historical records, 13 large floods occurred in Wuxi from 1983 to 2006 [24]. Simultaneously, Wuxi is subject to periodic, but not continuous, flooding and year-round high water tables. This is attributed to the specific geographical position and the local climatological characteristics [37]: (1) the river network is highly complicated, with a density of 3–4 km/km^2^; (2) a low-lying topography and numerous double-flow rivers exist in the network with limited draining and self-purification capacity; (3) the average annual precipitation is 674.70 mm from May to September, accounting for 64.38% of the annual rainfall. The Wuxi government launched large-scale flood control projects, and more than 800 floodgates of different sizes have been built to control flooding. Due to the urbanization process and the requirement for flood control, USD 0.17 billion will be invested to implement flood control emergency projects in Wuxi in the next decade. Therefore, the construction of these new floodgates and dams and the operation of existing ones will directly affect the water environment of Wuxi and Jiangsu Province, and even the Taihu Lake Basin.

Currently, about 50% of rivers in Wuxi have failed to meet the requirements of Class III of the “Water Environmental Quality Standards of China” (WEQSC). Among them, those in Wuxi’s downtown are the most seriously polluted, and the main pollution indicators are ammonia nitrogen (NH_3_-N), the permanganate index (COD_Mn_), the 5-day biochemical oxygen demand (BOD_5_), and total phosphorus (TP) [37]. The pollution sources in Wuxi are mainly from municipal solid waste, industrial pollution, and agricultural irrigation and fertilization. In this study, we selected 16 major rivers, with a total length of 291.34 km (Figure 1). Among them, the water quality of Wangyu River, Zhihugang River, Baiqugang River, Daxigang River, Li River, Bodugang River, the Xibei Canal, Jiuli River, Liangxi River, and Liangtang River is required to meet Class III of WEQSC (NH_3_-N ≤ 1.0 mg·L^−1^; COD_Mn_ ≤ 6 mg·L^−1^). Meanwhile, the water quality of the Xicheng Canal, the Beijing-Hangzhou Grand Canal, Xingou River, Yangxi River, Beixingtang River, and the Ancient Canal is required to meet Class IV (COD_Mn_ ≤ 10 mg·L^−1^; NH_3_-N ≤ 1.5 mg·L^−1^) (GB3838-2002).

A new “Flood Control Plan of Wuxi” was issued at the end of 2001, which would protect a 136 km^2^ region. In May 2003, the construction of flood control facilities began and was completed at the end of 2008. This project included seven flood control stations: Yandaigang flood control station, Beixingtang flood control station, Jiuli River flood control station, Bodugang flood control station, Limin Bridge flood control station, Xianli Bridge flood control station, and Jiangjian flood control station. These stations are operated not only to prevent floods in Wuxi but also to serve ship navigation. The floodgates are controlled by the relevant government departments according to the annual precipitation and total water quantity of Wuxi city.

## 3. Materials and Methods

### 3.1. Sampling and Experiment

(1) Sampling sites: The seven flood control stations were built on the rivers of Wuxi’s downtown: Xibei Canal, Beixingtang River, Jiuli River, Bodu River, Ancient Canal, Liangxi River, and Beijing-Hangzhou Grand Canal (Figure 1). In order to better analyze the relationship between the upstream and downstream of rivers and the location of the floodgates, avoiding the effect of a high flow velocity on the concentration of pollutants when the floodgates opened [39], the water sampling sites were installed 150–200 m upstream and downstream of the floodgates, totaling 14 sampling sites. Meanwhile, these monitoring sites have also been designated by the Jiangsu Environmental Monitoring Center since 2008.
(2) Sampling time: This study was conducted from January to December 2018, sampling in the middle of each month, including the wet and dry seasons. A suitable temperature was required to reduce the interference from external factors such as rainstorms on water quality. Meanwhile, when collecting water samples, water samples were taken 0.5–1.0 m below the water surface and far away from the river shore to reduce the impact of edge effects due to the shallow rivers [40]. All the floodgates were closed when collecting samples.
(3) Water quality indicators: COD_Mn_ and NH_3_-N are the most important indicators for compliance evaluation of water function zones in the Taihu Lake Basin, as well as the main monitoring indices of the Environmental Quality Standards for Surface Water (EQSSW) used in China [41,42]. Therefore, COD_Mn_ and NH_3_-N were selected as water quality indicators in this study. According to the national standards GB11892-89 and HJ535-2009, the detection of the COD_Mn_ concentration adopts the “Permanganate index method”, and that of NH_3_-N adopts “Gas-phase molecular absorption spectrometry”.
(4) Water sample processing: Before sampling, the river water was taken to clean and moisten the water extractor. Then, the polyethylene storage bottle was washed more than 3 times using the water in the water extractor, and the water sample was immediately taken full and placed into the cryogenic storage box for preservation. After sampling, all water samples were placed in the laboratory refrigerator at 4 °C, and all water quality data were measured within 24 h.

### 3.2. Data Sources

The pollution discharge data of 15 pollution sources and the monitoring data of 4 water quality monitoring points in this paper were obtained from the Wuxi Environmental Statistics and Wuxi Environmental Protection Bureau. The water quality monitoring points w1, w2, w3, and w4 are also the four main hydrological monitoring stations in Wuxi city. The hydrological data were sourced from the Wuxi Water Resources Bureau and the Wuxi Hydrological Bureau. The monitoring data from January to June 2018 were used for model calibration, and the data from July to December were used for model verification.

### 3.3. Methods

#### 3.3.1. Water Environment Model

##### Water Hydrodynamic Model

The hydrodynamic model (HD) can simulate various vertical homogeneous water flow conditions from steep mountainous rivers to tidal estuaries according to water flow conditions and subcritical flows in different regions. In addition, this model can also perform various simplified water flow simulations, such as the calculation of diffuse waves, motion waves, and quasi-steady flow [43].

The calculation parameters of HD include two types: ① numerical parameters, which are mainly the parameters related to the iterative solution of the system of equations, such as the number of iterations and the accuracy of the iterative calculation; ② physical parameters, mainly the resistance coefficient of the river network.

The governing equations of the one-dimensional hydrodynamic model are the Saint-Venant equations [43]:

(1) $$\left\{\begin{array}{l} \frac{\partial A}{\partial t}+\frac{\partial Q}{\partial x}=q\\ \frac{\partial A}{\partial t}+\frac{\partial }{\partial x}\left(\frac{Q^{2}}{A}\right)+gA\frac{\partial h}{\partial x}+g\frac{Q}{C^{2}}\frac{\left|Q\right|}{AR}=0 \end{array}\right.$$

where *t* is the time coordinate; *x* is the distance coordinate; *A* is the cross-section area; *Q* is the flow; *q* is the side inflow; *h* is the water level; *R* is the hydraulic radius; *C* is the riverbed roughness coefficient; and *g* is the gravity acceleration.

The hydrodynamic module, consisting of an implicit finite-difference 6-point Abbott–Ionescu scheme to solve the Saint-Venant equation, is the core part of the MIKE11 model. One of the additional modules is Structure Operations (SO), which is often used to define various structures in the river and their operating strategies, such as overflow gates, flood gates, radial gates, water pumps, and reservoir flood discharges. Through the SO module, any number of gates and dams can be selected to operate a variety of different dispatches. The MIKE11 model can simulate the water environment of a river network with different gates and dams while taking multiple objectives into account, including flood control and power generation.

##### Water Quality Model

The water quality model (AD), describing the transport of substances in the water column with a one-dimensional non-constant flow convective diffusion basic equation, is [44]

(2) $$\frac{\partial AC}{\partial t}+\frac{\partial QC}{\partial x}-\frac{\partial }{\partial x}\left(AD\frac{\partial C}{\partial x}\right)=-AKC+C_{2}q$$

where *C* is the concentration of the simulated water quality index; *D* is the diffusion coefficient; *Q* is the flow; *A* is the cross-sectional area; *K* is the comprehensive attenuation coefficient; and *q* is the side inflow.

There are two parameters to be determined in the water quality equation, namely, the diffusion coefficient *D* and the integrated attenuation coefficient *K*. The diffusion coefficient is an important parameter that reflects the longitudinal mixing characteristics of a river, and it is mainly affected by the flow conditions, cross-sectional characteristics, and rivers’ channel shapes. The comprehensive attenuation coefficient of pollutants is a comprehensive description of the physical, chemical, and biochemical reaction processes of pollutants in water bodies, including complex reaction processes such as river self-purification, sedimentation, and adsorption.

##### River Network and Boundary Conditions

**Figure 2:**
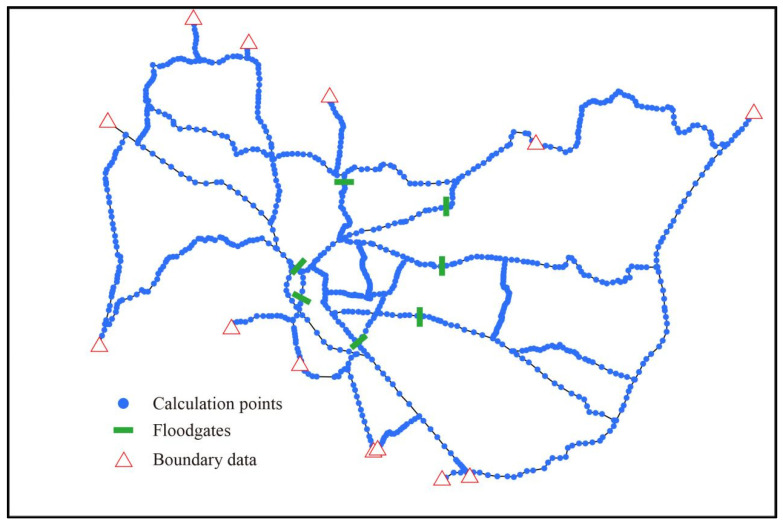
Generalization of the river network.

In this study, 16 major rivers in Wuxi were simulated and generalized into 52 branches and 1455 river cross-sections based on the topographic conditions and river channels in the city. Among them, 485 cross-sections were defined as flood control cross-sections (Figure 2). The rivers with a small volume and little impact on the water quality of the river network or the rivers with a narrow surface were not considered. The information data of all sluices were inserted into the SO module of the model (Figure 2). The river cross-section and longitudinal profile data were measured in 2004 and updated in 2010 based on the operational status of the flood control project. Compared with previous studies, this model can simulate the flood control process of the river network more accurately [42].

Considering the stability of the model and to reduce the error of the initial conditions of the model, the calculation period is from 00:00 on 1 January to 23:59 on 31 December, the calculation time of the model is set to 365 days, and the time step of the model is 1 s. In the HD model, the daily flow data of the river are selected as the boundary condition for the upper boundary, and the daily water level data are selected as the boundary condition for the lower boundary. In the AD model, monthly water quality monitoring data are set as the boundary conditions.

#### 3.3.2. Verification Model

To estimate the accuracy of the water environment model, the skill score (*SI*) and root mean square error (*RMSE*) between the simulated and observed data were chosen as validation parameters in this study. The *RMSE* indicates the degree of distinction between the simulated and observed data, and the *SI* describes the similarity in their spatial distribution. The values of *SI* range from 0 to 1, representing “poor simulation results” and “perfect match”, respectively. They are defined as [24]

(3) $$SI=\frac{\sum_{i=1}^{n}\left(O_{i}\times S_{i}\right)}{\sqrt{\sum_{i=1}^{n}{\left(O_{i}\right)}^{2}}\times \sqrt{\sum_{i=1}^{n}{\left(S_{i}\right)}^{2}}}$$

(4) $$RMSE=\sqrt{\frac{1}{n}\sum_{i=1}^{n}{\left(O_{i}-S_{i}\right)}^{2}}$$

where *n* is the water quality monitoring points, and *O_i_* and *S_i_* are the observed data and the simulated data, respectively.

## 4. Calibration and Verification of Water Environment Model

The length of the stable computation time for the model was set to 365 days, which could reduce the model bias in the initial field of the model. This study used trial-and-error methods to calibrate the HD and AD modules based on hydrological data of 2018. The diffusion coefficient of the river network was 15 m^2^·s^−1^. All parameters were the same in the simulations with and without gates, except for the pollutant degradation coefficient. The pollutant degradation coefficient is one of the most important parameters in the AD module. The rate determination results were obtained according to the recommended values by other scholars [41,42] (Table 1).

**Figure 3:**
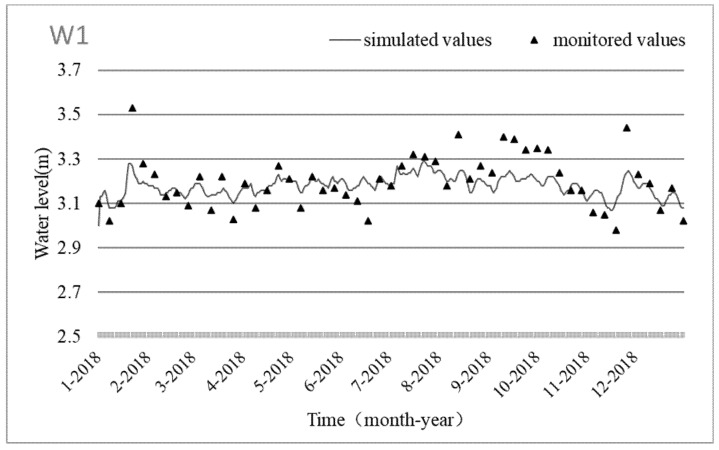

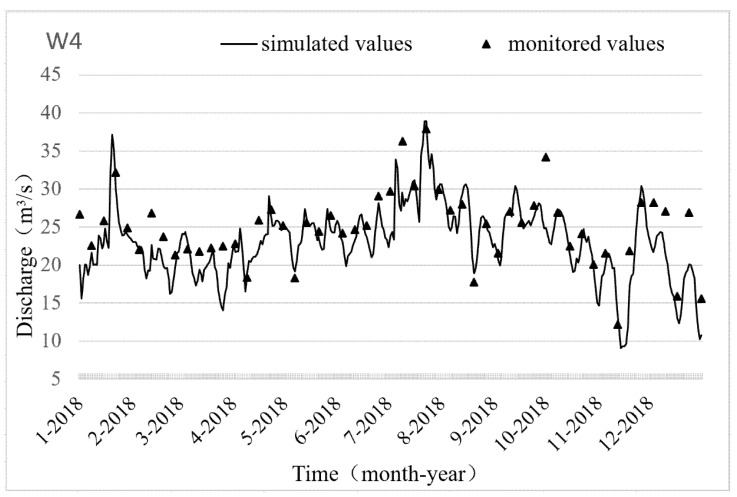
Comparison of simulated and monitored values of water levels and discharges.

During the calibration and validation of the hydrodynamic model, the relative errors of flow at the four hydrological stations were less than 0.30, and the relative errors of the water level were less than 0.11. It was found that the model simulation results were acceptable and could accurately reflect the hydrodynamic change process. Further modeling work could be carried out. The monitored values of the water level at monitoring point w1 and flow at w4 are compared with the simulated values in Figure 3.

Water quality data from four water quality monitoring sites were applied to the calibration and validation of the water quality model. The simulation results showed a logical quantitative and qualitative coordination between the simulated and monitored concentrations (Table 2). The *RMSE* ranged from 0.23 to 1.65 with an average of 0.94, and the *SI* ranged from 0.81 to 0.95 with an average of 0.89. According to the above analysis statistics, the model presented satisfactory simulation results compared to the monitoring results. Therefore, the next step of the simulation could be carried out.

## 5. Results and Discussion (Scenario Analysis of Water Quality Response to Floodgate Operation)

Wuxi’s rivers, like other urban rivers in China, often receive a portion of untreated urban domestic sewage. This tends to create large pollution masses and cause localized problems of poor water quality. When floodgates are closed, water quality tends to worsen because of the lack of dilution from upstream water or external sources [38]. According to the “Thirteenth Five-Year Plan” of Wuxi city and the Wuxi Water System Plan, the water quality of ten rivers in the river network must meet Class III of the surface water environmental quality standard (COD_Mn_ ≤ 6 mg·L^−1^; NH_3_-N ≤ 1.0 mg·L^−1^). The other six rivers must meet Class Ⅳ of WEQSC (COD_Mn_ ≤ 10 mg·L^−1^; NH_3_-N ≤ 1.5 mg·L^−1^). In addition, according to the Wuxi City Urban Flood Control Plan and Flood and Drought Control Guidelines, the maximum water level for flood prevention in Wuxi is 3.8 m, and the minimum water level for drought control is 2.8 m. Therefore, with the purpose of satisfying the water quality requirements of Wuxi’s river network and the requirements of flood and drought control, this study proposed three design solutions of floodgates, which were 0.7 m, 1.4 m, and 2.1 m floodgate heights above the water surface.

### 5.1. Analysis of Status Quo on Floodgate Operation

**Figure 4:**
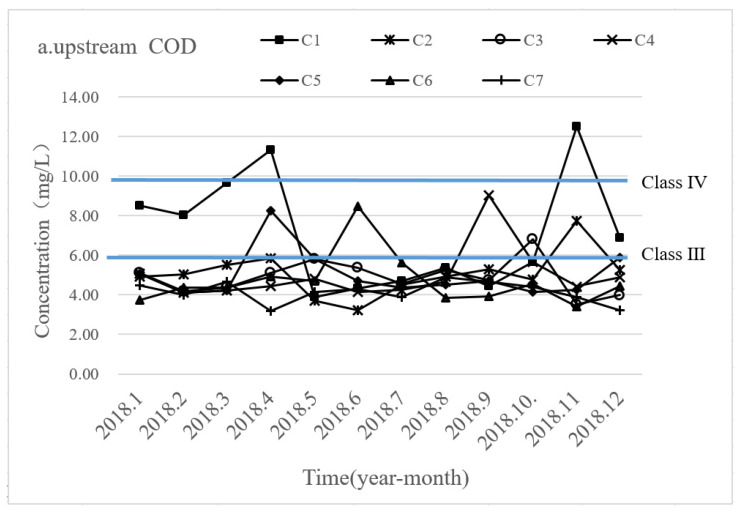

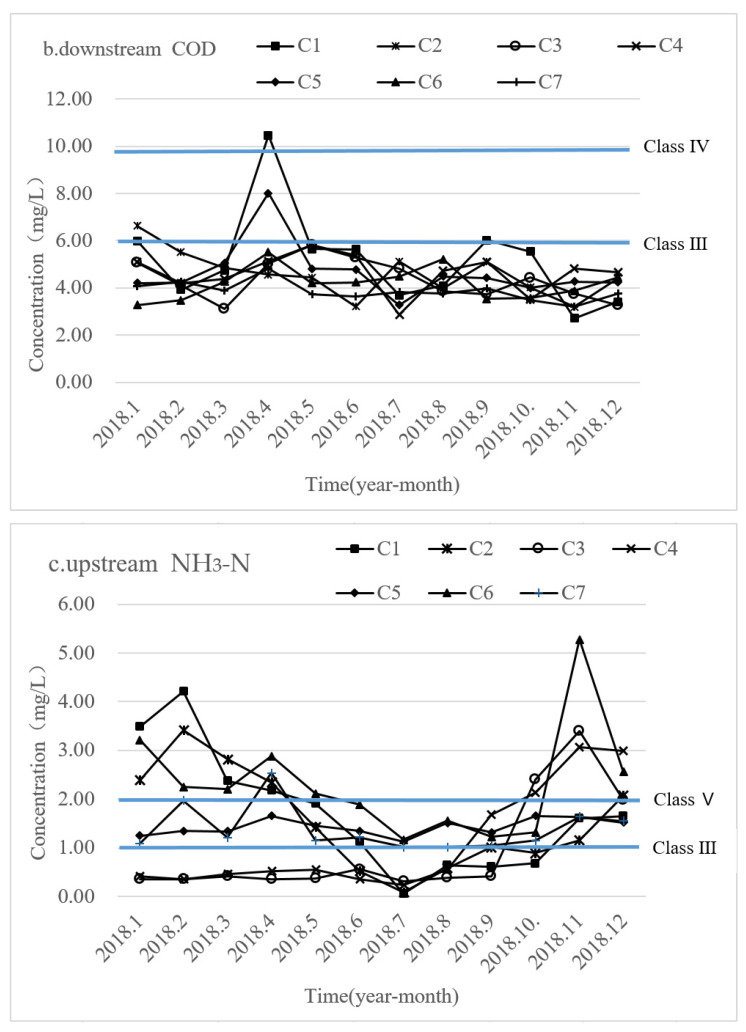

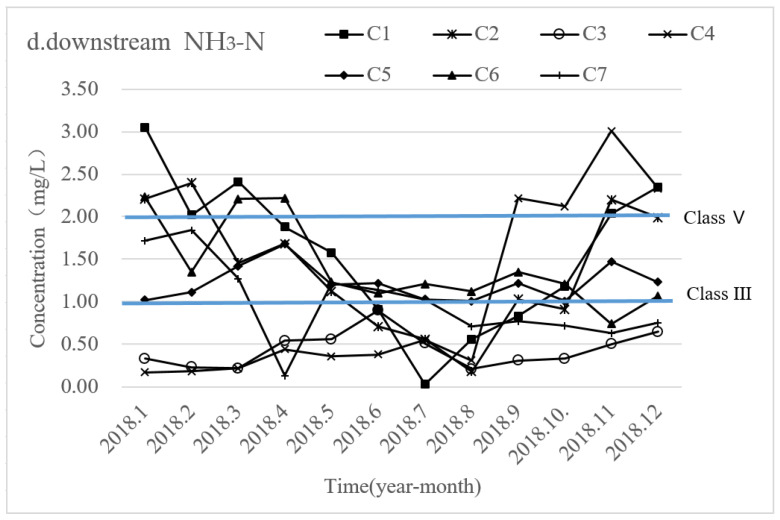
The changes in COD_Mn_ and NH_3_-N concentrations under the current floodgate operation. (**a**) COD_Mn_ concentration in the upstream; (**b**) COD_Mn_ concentration in the downstream; (**c**) NH_3_-N concentration in the upstream; (**d**) NH_3_-N concentration in the downstream.

In order to investigate the changes in the responses of COD_Mn_ and NH_3_-N under different design solutions, this study compared and analyzed the responses of the two pollutants to the status quo of the floodgate regulation (Figure 4). From the distribution of the pollutant concentrations before and after each floodgate, the COD_Mn_ concentration is basically in Class III of WEQSC. In the upstream area of floodgates, the COD_Mn_ concentrations at the four monitoring points of C2, C4, C5, and C6 exceeded Class III in April, June, September, and November, while the COD_Mn_ concentrations at monitoring point C1 exceeded Class IV in both April and November. In the downstream area, the COD_Mn_ concentration was generally lower than that in the upstream area. The COD_Mn_ concentrations at monitoring points C4, C5, and C1 exceeded Class III in January, April, and September, while the COD_Mn_ concentration at monitoring point C1 exceeded Class IV in April.

Compared with the COD_Mn_ concentration condition, the NH_3_-N concentration exceeded the standard more significantly. In the upstream area, NH_3_-N concentrations at monitoring points C1, C2, and C6 exceeded Class V of WEQSC from January to May. From October to December, the NH_3_-N concentrations at C3, C4, and C6 also exceeded Class V. In the downstream area, NH_3_-N concentrations at C1, C2, and C6 exceeded Class V from January to April. From November to December, the NH_3_-N concentrations at C1, C2, and C4 exceeded Class V of WEQSC. In general, during the dry period (October to February) and the flat period (March to May) of the river network, the pollutant concentration is in a relatively high state when the floodgates are closed, and the water quality of the river is relatively poor. This result is highly consistent with previous investigations [27,45], which found that water control projects (floodgates and dams) were forces to be reckoned with and would certainly impact the development of the whole water environment.

### 5.2. Analysis of Design Solutions on Floodgate Operation

#### 5.2.1. Solution No.1 (0.7 m)

**Figure 5:**
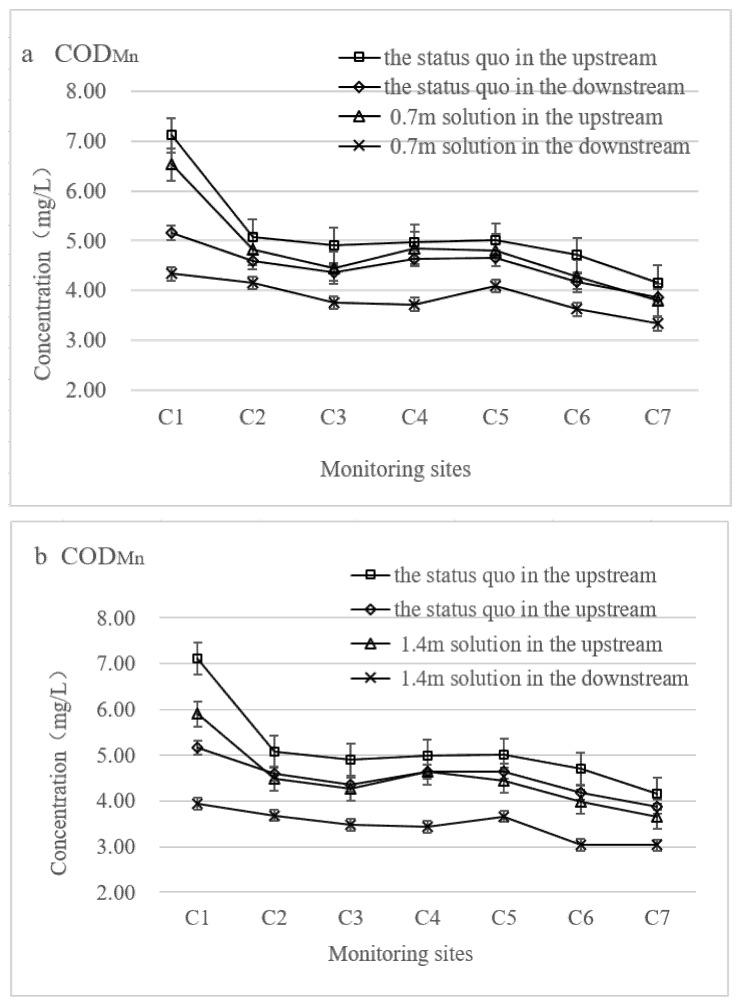

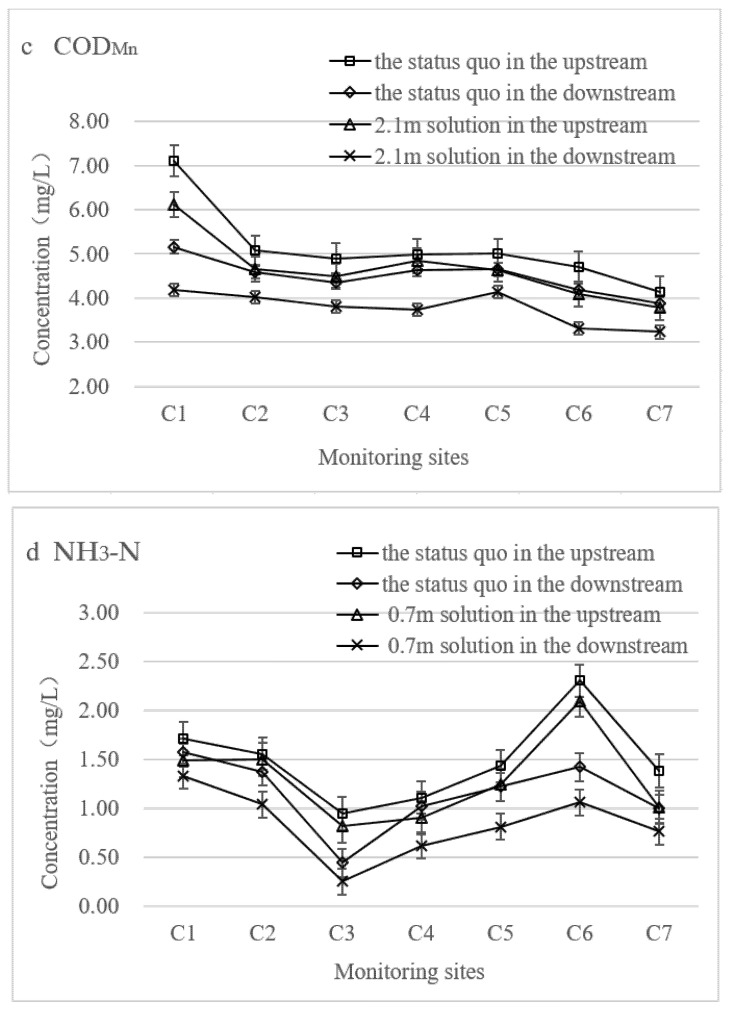

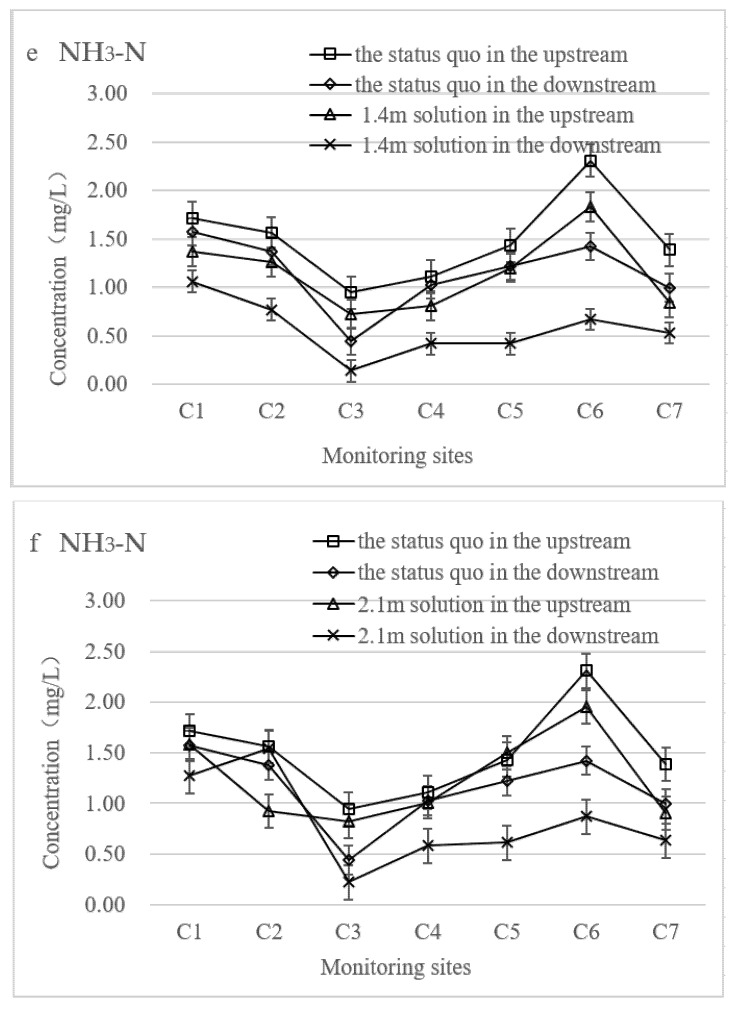
The changes in COD_Mn_ and NH_3_-N concentrations under different floodgate operations. (**a**) COD_Mn_ concentration changes under 0.7 m solution; (**b**) COD_Mn_ concentration changes under 1.4 m solution; (**c**) COD_Mn_ concentration changes under 2.1 m solution; (**d**) NH_3_-N concentration changes under 0.7 m solution; (**e**) NH_3_-N concentration changes under 1.4 m solution; (**f**) NH_3_-N concentration changes under 2.1 m solution.

When the floodgate height was adjusted to 0.7 m, the COD_Mn_ and NH_3_-N concentrations at all seven monitoring points showed a decreasing trend compared to the current situation of the floodgate regulation (Figure 5a,d). Among them, the average COD_Mn_ concentrations at monitoring points C3 and C6 decreased the most by 0.45 mg·L^−1^ and 0.43 mg·L^−1^, respectively, and the maximum decline rate for both was 10% in the upstream area. The average NH_3_-N concentration at monitoring site C7 decreased by 0.37 mg·L^−1^, and the maximum rate of decrease was 37%. The simulation results show that the water quality status of the river network under the floodgate regulation was better than that under the situation of floodgate closure.

In the downstream area, the advantages of water quality improvement caused by the floodgate regulation were more obvious. The average COD_Mn_ concentrations of the seven monitoring sites fell by more than 10%. Among them, the average COD_Mn_ concentrations at monitoring points C1 and C4 decreased by 0.83 mg·L^−1^ and 0.91 mg·L^−1^, and the decrease rates were 19.09% and 24.5%, respectively. The average NH_3_-N concentrations at all seven monitoring sites decreased by more than 17%. Among them, the average NH_3_-N concentrations at the C3 and C4 monitoring sites decreased by 0.19 mg·L^−1^ and 0.40 mg·L^−1^, and the decrease rates were 78% and 64.75%, respectively. The comparison of the upstream and downstream simulation results showed that the water quality condition in the downstream area of the river network was improved more significantly under the floodgate regulation. This phenomenon could be attributed to the fact that these floodgates were frequently operated for flood control in summer, which accelerated the renewal of the water mass [24].

#### 5.2.2. Solution No.2 (1.4 m)

When the floodgate height was adjusted to 1.4 m, the COD_Mn_ and NH_3_-N concentrations showed different degrees of decrease at the seven monitoring points at the same time (Figure 5b,e). In the upstream area, the average COD_Mn_ concentration at monitoring site C4 decreased the least, with a decrease of 0.35 mg·L^−1^, and the rate of decline was 8%. The largest decrease in the COD_Mn_ concentration was observed at monitoring site C1, with a reduction of 1.21 mg·L^−1^, and the rate of decline was 21%. Compared to COD_Mn_, the changes in NH_3_-N concentrations were more prominent. The average NH_3_-N concentrations at the seven monitoring sites all decreased by more than 19%. The average concentration of NH_3_-N at the C5 monitoring point decreased by 0.23 mg·L^−1^, and the rate of decline was 19.32%. The average concentration of NH_3_-N at monitoring point C7 decreased by 0.54 mg·L^−1^, and the rate of decline was 64%. It can be seen that the adjusted height of the floodgates had a great influence on the concentration of pollutants in the river network. This result is in accordance with the control measure efficiency analysis in Guo River, China, in which the water quality was improved by floodgate operation [35].

In the downstream area, the average COD_Mn_ concentrations at all seven monitoring sites decreased by more than 25%. Among them, the average COD_Mn_ concentrations at monitoring points C4 and C6 were reduced by 1.2 mg·L^−1^ and 1.14 mg·L^−1^, and the rates of decline were 35.11% and 37.57%, respectively, accounting for the largest proportion. In the downstream region, NH_3_-N concentrations significantly decreased, and their changes obviously exceeded those in the COD_Mn_ concentrations. The mean NH_3_-N concentrations at monitoring sites C2 and C7 decreased by 0.6 mg·L^−1^ and 0.47 mg·L^−1^, and the rates of decline were 78% and 88%, respectively. At monitoring points C3, C4, C5, and C6, the average NH_3_-N concentrations decreased by 206%, 145%, 188%, and 111%, respectively. In this solution, the comparison of the upstream and downstream simulation results showed that the improvement in water quality in the downstream area of the river network was significantly better than that in the upstream area under the floodgate regulation. In addition, the water quality improvement under the solution of the adjusted floodgate height of 1.4 m was significantly better than that at the height of 0.7 m.

#### 5.2.3. Solution No.3 (2.1 m)

When the floodgate height was adjusted to 2.1 m, the average concentrations of COD_Mn_ and NH_3_-N still showed a decreasing trend, as shown in Figure 5c,f. In the upstream area, the average COD_Mn_ concentration at the C4 monitoring site decreased the least, with a decrease of 3%. The average COD_Mn_ concentration at monitoring point C1 decreased the most, with a decrease of 16%. Unlike the change in the average COD_Mn_ concentration, the average NH_3_-N concentration showed an increasing trend at the C5 monitoring point, increasing by 0.06 mg·L^−1^, and the rate of increase was 4.29%. At the same time, the average NH_3_-N concentrations of other monitoring points showed a downward trend, with a maximum decrease of 54% at the C7 monitoring point. Overall, the floodgate regulation indeed improved the water quality condition of the river network.

In the downstream area, the concentrations of the two pollutants continued to show a decreasing trend. The average COD_Mn_ concentration decreased by 0.51 mg·L^−1^ at monitoring point C5, and the decreasing rate was 12%. The concentration at the C6 monitoring point decreased by 0.87 mg·L^−1^, and the largest drop was 26.23%. Compared to COD_Mn_, the average NH_3_-N concentration decreased more significantly. At monitoring points C6, C4, C5, and C3, the average NH_3_-N concentrations decreased by 63.69%, 75.90%, 100%, and 102%, respectively. From the simulation results, it can be seen that the water quality in the downstream area of the river network was better than that in the upstream area under the floodgate regulation. At the same time, the water quality improvement under the 2.1 m scenario of the floodgate regulation was worse than that under the 1.4 m scenario of the regulation.

### 5.3. Seasonal Analysis on Floodgate Operation

**Figure 6:**
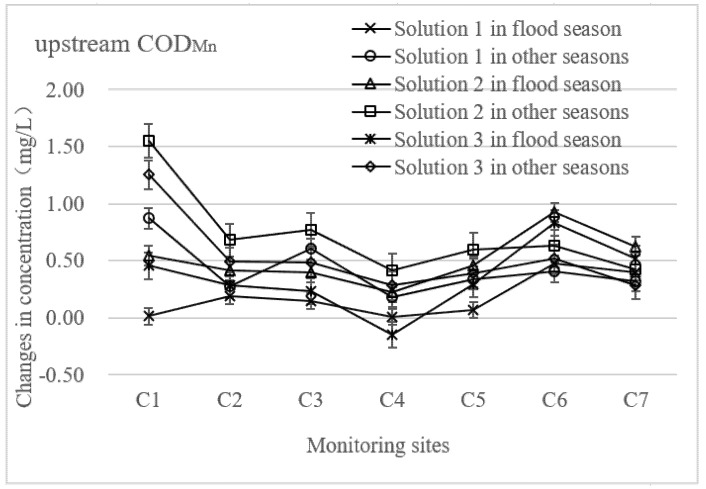

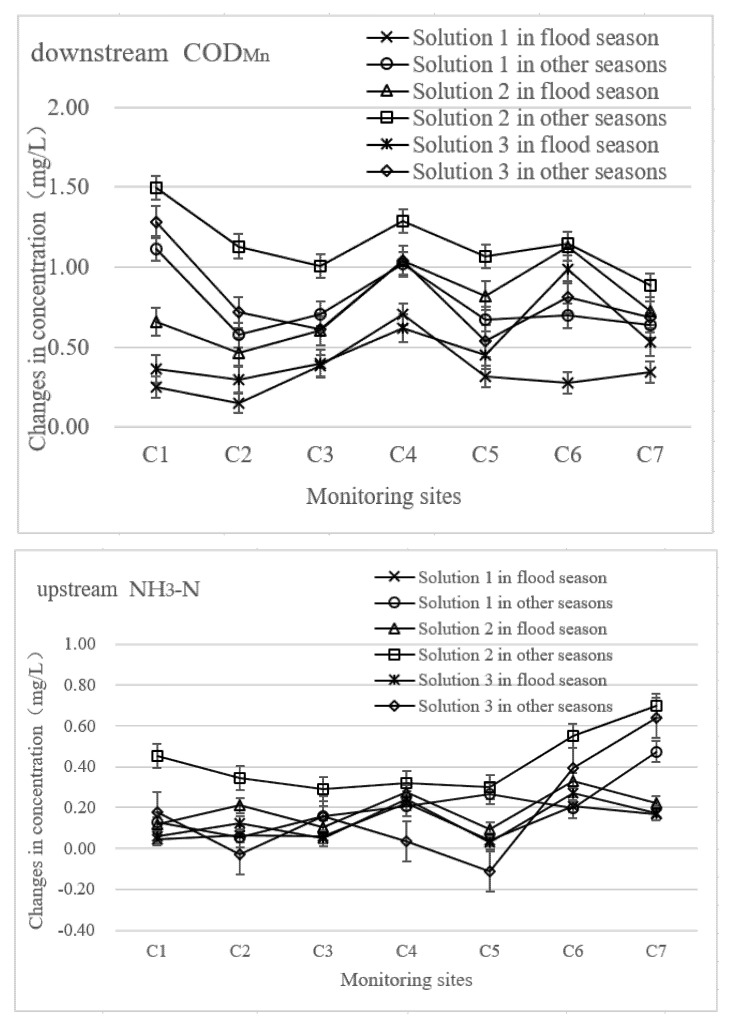

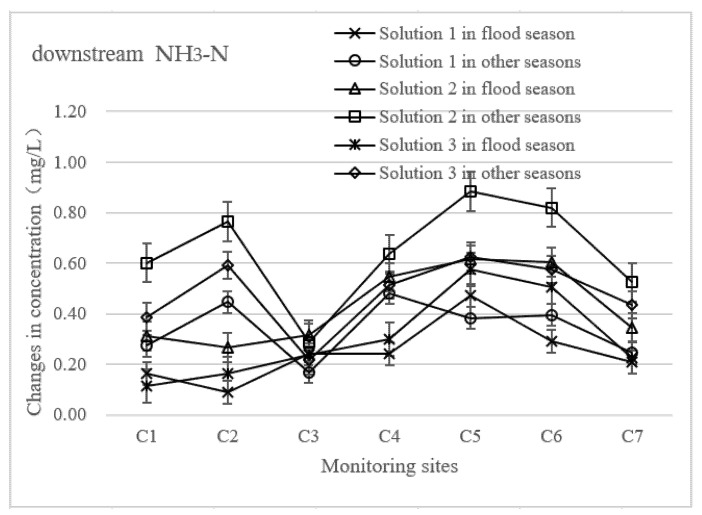
The seasonal changes in COD_Mn_ and NH_3_-N concentrations under different floodgates.

In addition, a full-year analysis of the three design solutions of the floodgate regulation showed that from June to September, i.e., the flood season in Wuxi, the variation levels of the two pollutants’ concentrations were significantly lower than those during the dry season (October to February) and the flat season (March to May) in one year (Figure 6).

The variation in the COD_Mn_ concentration was 0.12 mg·L^−1^ from June to September in the upstream region under solution No.1 of the floodgate regulation, while the variation value was 0.67 mg·L^−1^ in the other months. In the same situation, the change value of the NH_3_-N concentration was 0.06 mg·L^−1^, and the change value was 0.16 mg·L^−1^ in the other months. In the downstream region, COD_Mn_ concentrations varied at 0.15 mg·L^−1^ during the flood period, while the variation value was 0.72 mg·L^−1^ in the other months. This result shows that the implication of the floodgate regulation on the water quality in the river network cannot be ignored. During the annual flood season in Wuxi, the floodgates are regulated by the water conservancy department. The floodgates are opened at the right times according to the water level changes to prevent flooding, while in the dry and flat seasons, the floodgates remains closed for a long time. This explains why the concentrations of the two pollutants in the regulation scenarios vary little in the flood season, but a lot in the other months. Additionally, in the river network, the temperature of the water body, water quality in the upstream, and atmospheric pressure are also possible factors that influenced the COD_Mn_ and NH_3_-N concentrations [38]. In summer, the average temperature is 80 °F, and the maximum is 102 °F. A higher temperature could accelerate the decomposition of organic matter in the sediment, which causes more NH_3_-N to be released in rivers [46].

Compared to solution No.1, the changes in the concentrations of the two pollutants are more obvious in scenario No.2. In the upstream region, the variation value of the COD_Mn_ concentration was 0.41 mg·L^−1^ in the flood season and 0.82 mg·L^−1^ in the other months. The change in the NH_3_-N concentration was 0.11 mg·L^−1^ during the flood period and 0.29 mg·L^−1^ during the other months. In the downstream area, the change in the COD_Mn_ concentration was 0.45 mg·L^−1^ from June to September and 0.92 mg·L^−1^ in the other months. The change in the NH_3_-N concentration was 0.15 mg·L^−1^ from June to September and 0.40 mg·L^−1^ in the other months. It can also be seen that the floodgate regulation had a greater impact on the variation in pollutant concentrations in the downstream regions.

Comparing the three scenarios, the concentration of the two pollutants changed more obviously in the downstream area under the 1.4 m scenario of the floodgate regulation, especially in the dry season and the flat season.

## 6. Conclusions

This paper established a hydrodynamic–water quality coupling model in the river network area of Wuxi city. The migration and transformation rules of COD_Mn_ and NH_3_-N were analyzed, and the model was validated by the measured hydrological and water quality data from 2018. The simulation results demonstrate that the model had high accuracy in the hydrodynamic–water quality simulation process, could simulate the hydrodynamic conditions of the Wuxi river network well, and obviously reflected the changes in water quality. It is suitable for the prediction and simulation analysis of future small floodgate and dam construction.

Among the three design solutions, the reductions in the COD_Mn_ and NH_3_-N concentrations were ranked as follows: 1.4 m solution of the floodgate regulation > 2.1 m solution of the floodgate regulation > 0.7 m solution of the floodgate regulation. Under the 1.4 m solution, the maximum decrease in the COD_Mn_ concentration reached 37.57%, the maximum decrease in the NH_3_-N concentration reached 206%, and the status of the water quality in the downstream area of the river network was significantly better than that in the upstream area.

This paper simulated different design solutions of the floodgate regulation according to the actual situation of the construction and operation of floodgates and dams in the Wuxi river network. This study obtained different degrees of improvement in pollutant concentrations. The results of this paper can supply new ideas for the management of polluted rivers in other multi-floodgate areas and for the construction and operation of new floodgates and dams.

It should be noted here that we analyzed just two pollutants in terms of water quality (COD_Mn_ and NH_3_-N) under three different scenarios. Other pollutants were not studied as parameters in this paper. Due to the lack of indispensable historical data, this study did not investigate the impact of floodgates on river ecosystems, and thus further ecological analysis is necessary.

## Figures and Tables

**Table 1:** Degradation coefficient for the quality of pollution in the river network (unit: d^−1^).

	Scenario with Floodgates		Scenario without Floodgates	
	COD_Mn_	NH_3_-N	COD_Mn_	NH_3_-N
Beijing-Hangzhou Grand Canal	0.18	0.09	0.12	0.04
Baiqugang River	0.12	0.05	0.08	0.03
Beixingtang River	0.15	0.08	0.11	0.07
Jiuli River	0.16	0.07	0.13	0.05
Bodugang River	0.11	0.08	0.08	0.06
Daxigang River	0.13	0.07	0.12	0.06
Liangxi River	0.15	0.09	0.11	0.08
Ancient Canal	0.18	0.09	0.12	0.05

**Table 2:** Simulation results of water quality monitoring points.

	Parameters	*RMSE*		*SI*	
Monitoring Sites		COD_Mn_	NH_3_-N	COD_Mn_	NH_3_-N
1		1.65	0.65	0.94	0.95
2		1.32	0.22	0.92	0.79
3		1.21	1.34	0.92	0.88
4		0.91	0.23	0.93	0.81

## Data Availability

Not applicable.
